# Beyond the Black Box: Physics-Based and Conservation-Informed Evidence for ACVRL1 Variant Classification in Hereditary Hemorrhagic Telangiectasia and Pulmonary Arterial Hypertension

**DOI:** 10.3390/ijms27219742

**Published:** 2026-10-30

**Authors:** Klaus Brusgaard

**Affiliations:** 1 Department of Clinical Genetics, Vejle Hospital, Sygehus Lillebælt, Beriderbakken 4, 7100 Vejle, Denmark; klaus.brusgaard@rsyd.dk; 2 Department of Regional Health Research, Institute of Health, University of Southern Denmark, Campusvej 55, 5230 Odense, Denmark

**Keywords:** ACVRL1, ALK1, hereditary hemorrhagic telangiectasia, pulmonary arterial hypertension, variant classification, missense variant, protein structure, conservation, interpretable machine learning

## Abstract

Missense variants in ACVRL1 (ALK1) cause hereditary hemorrhagic telangiectasia type 2 (HHT2), heritable pulmonary arterial hypertension (PAH), or both in the same patient, and many remain variants of uncertain significance. AlphaMissense classifies them accurately but does not show which evidence drives a call. I annotated 899 ACVRL1 missense variants with protein-stability estimates (FoldX, DynaMut2), a GREMLIN-style conservation score, and structural context. I then trained random-forest classifiers on 311 variants with labels traceable to ClinVar or a publication (296 pathogenic-like, 15 benign-like). A structural/conservation-only classifier (Model 1b) reached a cross-validated ROC-AUC of 0.954 (95% CI 0.916–0.984), and a model adding sequence-based predictors (Model 1) reached 0.979, versus 0.983 for AlphaMissense on the same variants. On variants first reported in 2025–2026, Model 1b scored 0.976, Model 1 0.992, and AlphaMissense 0.992; equivalence within ±0.05 AUC was supported for Model 1 only. Benign variants were the main weakness: Model 1b correctly classified 10/15, against 14/15 for AlphaMissense. Conservation drove most calls, with predicted destabilization contributing to a minority. An interpretable, gene-specific classifier can approach AlphaMissense for ACVRL1 while showing the structural or evolutionary evidence behind each call. Its calls are hypotheses, not functional evidence, and can prioritize VUS for functional testing.

## 1. Introduction

ACVRL1 encodes activin receptor-like kinase 1 (ALK1), an endothelial-cell-specific type I receptor of the transforming growth factor-β (TGF-β) superfamily. Together with the co-receptor endoglin (ENG) and the type II receptor BMPR2, ALK1 forms a complex activated by the circulating ligands BMP9 and BMP10. This complex triggers SMAD1/5/9 phosphorylation and downstream transcriptional control of vascular quiescence genes [1]. Heterozygous loss-of-function variants in ACVRL1 cause hereditary hemorrhagic telangiectasia type 2 (HHT2), one of the two major genetic subtypes of HHT. The other, HHT1, is caused by ENG variants, and together they account for the large majority of clinically diagnosed HHT [1]. A separate, clinically distinct phenotype, heritable pulmonary arterial hypertension (PAH), is also caused by ACVRL1 variants. It is disproportionately associated with variants in the intracellular kinase domain, consistent with a partial loss-of-function or hypomorphic mechanism rather than the simple haploinsufficiency more typical of HHT2 [2,3].

At the genetic level, HHT has classically been explained by haploinsufficiency: a single heterozygous loss-of-function allele halves the amount of functional ALK1 or endoglin protein throughout the vasculature. This model does not explain why vascular malformations are focal. Snellings et al. found low-frequency somatic second-hit variants in ENG or ACVRL1 within HHT telangiectasias, in trans to the germline variant, supporting a two-hit mechanism [4]. A larger study of arteriovenous malformations [5] and deep sequencing of several lesions from one patient with a germline ACVRL1 deletion [6] confirmed this pattern, and the topic was recently reviewed [7]. The germline variant is therefore necessary but not sufficient for each lesion. Accurate germline classification remains the basis for diagnosis, cascade testing, and counseling, but it does not predict where or when a malformation will arise.

Missense variants dominate the ACVRL1 mutational spectrum and account for most variants of uncertain significance (VUS) in this gene. Under the ACMG/AMP framework [8], in silico predictors such as SIFT [9], PolyPhen-2 [10], REVEL [11], and CADD [12] contribute supporting computational evidence. AlphaMissense, a deep-learning model built on structure prediction and population data, outperforms these earlier tools and approaches expert-level concordance with curated databases [13]. Like other deep-learning predictors, however, it returns a single probability without indicating which physical or evolutionary property drove the call. Two recent studies suggest that this gap can be closed. Ramadane-Morchadi et al. showed that a gene-specific structural score can refine the PP3/BP4 criteria for BRCA1 [14]. Banerjee et al. introduced Rhapsody-2, which combines coevolution, structure, and short-timescale dynamics into a mechanistic score [15]; I benchmark against it below (Section 2.9).

The purpose of this work is therefore not to replace AlphaMissense but to add what a single probability cannot show. For each variant, the classifier shows the residue-level evidence behind its call: a lost disulfide bond, a destabilized fold, a BMPR2- or FKBP1A-interface position, or an invariant residue on a stable surface. This study has two aims. First, I build a classifier for ACVRL1 missense variants from protein-stability, structural, and evolutionary-conservation evidence only, without population-frequency data, and compare it with AlphaMissense on the same variants. A companion classifier adds standard sequence-based predictors (REVEL, CADD, SIFT, PolyPhen, phyloP) to measure what they contribute. Second, as an exploratory aim, I test whether structural and positional features alone separate variants reported in PAH from those reported in HHT.

## 2. Results

### 2.1. Variant Assembly and Data-Integrity Quality Control

The final dataset comprises 899 ACVRL1 missense variants (Table S1): 886 from the original structural/conservation screen plus 13 additions found through a full-text PubTator3 literature sweep (12 variants; Table S2) and one individually cited case report (A347G). A post hoc check of each added variant’s wild-type residue against UniProt P37023 identified three problems among an initial 16 candidate additions. S239C and C382W were dropped (positions 239 and 382 are tryptophan and aspartate; C382W traced to wrong-gene contamination during literature mining), and L306P was corrected to A306P, matching the source paper’s HGVS table. A306P duplicated a variant already in the screen, so its citation and functional-assay result were merged into the existing row. ClinVar classifications agreed with a fresh ClinVar download for 320 of 323 variants (99.1%), and REVEL scores were reproducible (r = 1.000 across 474 variants).

Every training label was then traced to a documented source (Table S3). Of 315 candidate labels, 104 initially lacked a recorded source. Sixty of these were ClinVar entries whose classification had not been carried into the table; 58 were confirmed against ClinVar, with the VCV accession recorded, and none changed class. For 11 further labels whose ClinVar classification had not been recorded, the current ClinVar classification was retrieved: all 11 are pathogenic (P378L pathogenic/likely pathogenic, 1–2 submitters each), in agreement with the training labels (Table S3). Forty-four published case reports were identified with the help of HGMD Professional (release 2026.3). I reviewed each primary publication and kept a label only where the authors reported the variant as disease-causing in an affected individual: 41 were confirmed with a PMID and one through a non-indexed journal article. E281Q and H66P were reclassified as VUS (single reports without segregation or functional data). G211A was removed because no publication could be retrieved, and L135R because it has no ClinVar record and its population frequency alone does not meet BA1. The final training set comprises 311 variants (296 pathogenic-like, 15 benign-like) on 158 residues, each with a named ClinVar record or publication. No HGMD classifications or accession numbers are reproduced in the dataset.

### 2.2. Classifier Performance Compared with AlphaMissense

Two random-forest classifiers were trained on the 311 labeled variants. Model 1b uses only structural and conservation evidence: FoldX and DynaMut2 ΔΔG, the GREMLIN-style conservation ΔΔE, ortholog conservation statistics, AlphaFold3 [16] pLDDT, IUPred3 disorder, secondary structure, and structural-interface membership. It uses no population-frequency or sequence-based predictor input. Model 1 adds REVEL, CADD, SIFT, PolyPhen, phyloP, and gnomAD allele frequency. Molecular-dynamics and electrostatic features were not included in either model because an ablation showed that they added no predictive value (Section 2.6, Table S4). Under 5-fold stratified cross-validation (random_state = 42), Model 1b achieved ROC-AUC 0.954 (95% bootstrap CI 0.916–0.984) and Model 1 achieved 0.979 (0.957–0.994); AlphaMissense scored 0.983 (0.962–0.997) on the same variants. Across 20 random seeds the means were 0.947 ± 0.008 for Model 1b and 0.976 ± 0.004 for Model 1. Because the 311 substitutions fall on 158 residues, I also kept all substitutions at a residue in the same fold (residue-grouped cross-validation, five seeds); performance fell slightly to 0.937 ± 0.009 and 0.972 ± 0.004.

**Figure 1:**
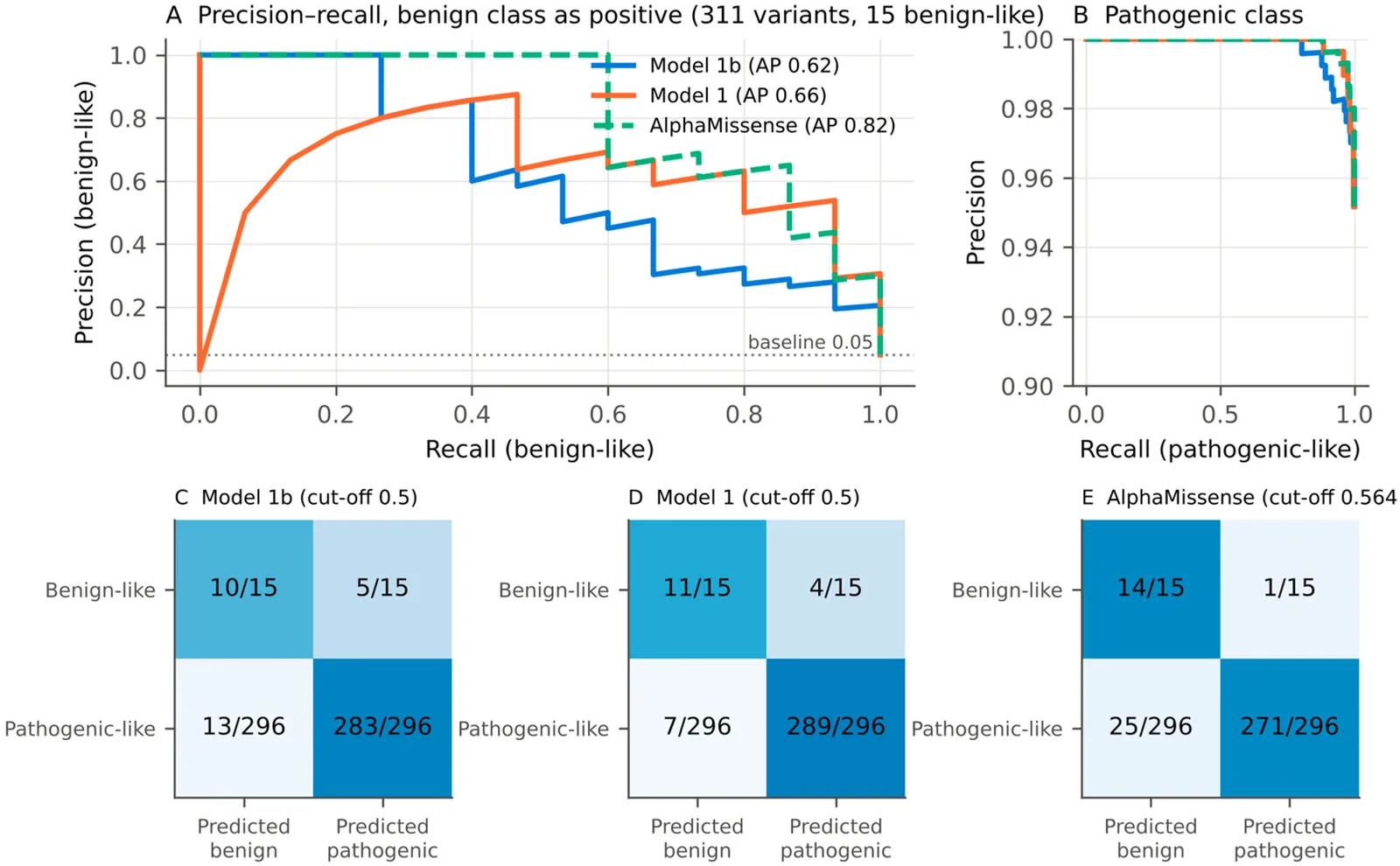
Imbalance-aware evaluation on the 311 labeled variants (out-of-fold predictions for Models 1b and 1). (**A**) Precision–recall curves with the benign class as positive; dotted line = baseline prevalence (0.05). (**B**) Precision–recall with the pathogenic class as positive. (**C**–**E**) Confusion matrices at the 0.5 threshold for Models 1b and 1 and at the published 0.564 cut-off for AlphaMissense. Line colors in (**A**,**B**) identify the predictors (legend); in (**C**–**E**), cell shading is proportional to the fraction of each true class.

With only 15 benign-like variants (4.8%), ROC-AUC alone can hide a substantial benign error rate, so Table 1 and Figure 1 report imbalance-aware metrics. Average precision for the benign class was 0.62 for Model 1b, 0.66 for Model 1, and 0.82 for AlphaMissense, against a baseline of 0.05. At the pre-specified 0.5 threshold, Model 1b correctly classified 10/15 benign variants (95% CI 0.42–0.85) and 283/296 pathogenic variants (balanced accuracy 0.81, MCC 0.51). Model 1 classified 11/15 and 289/296 correctly (balanced accuracy 0.85). AlphaMissense at its published cut-off (0.564) classified 14/15 benign and 271/296 pathogenic variants correctly (balanced accuracy 0.92). With a threshold chosen inside each training fold, Model 1b recognized 12/15 benign variants at a pathogenic sensitivity of 263/296, and Model 1 14/15 at 271/296. Benign recognition is therefore the weakest property of both models, particularly of Model 1b.

**Figure 2:**
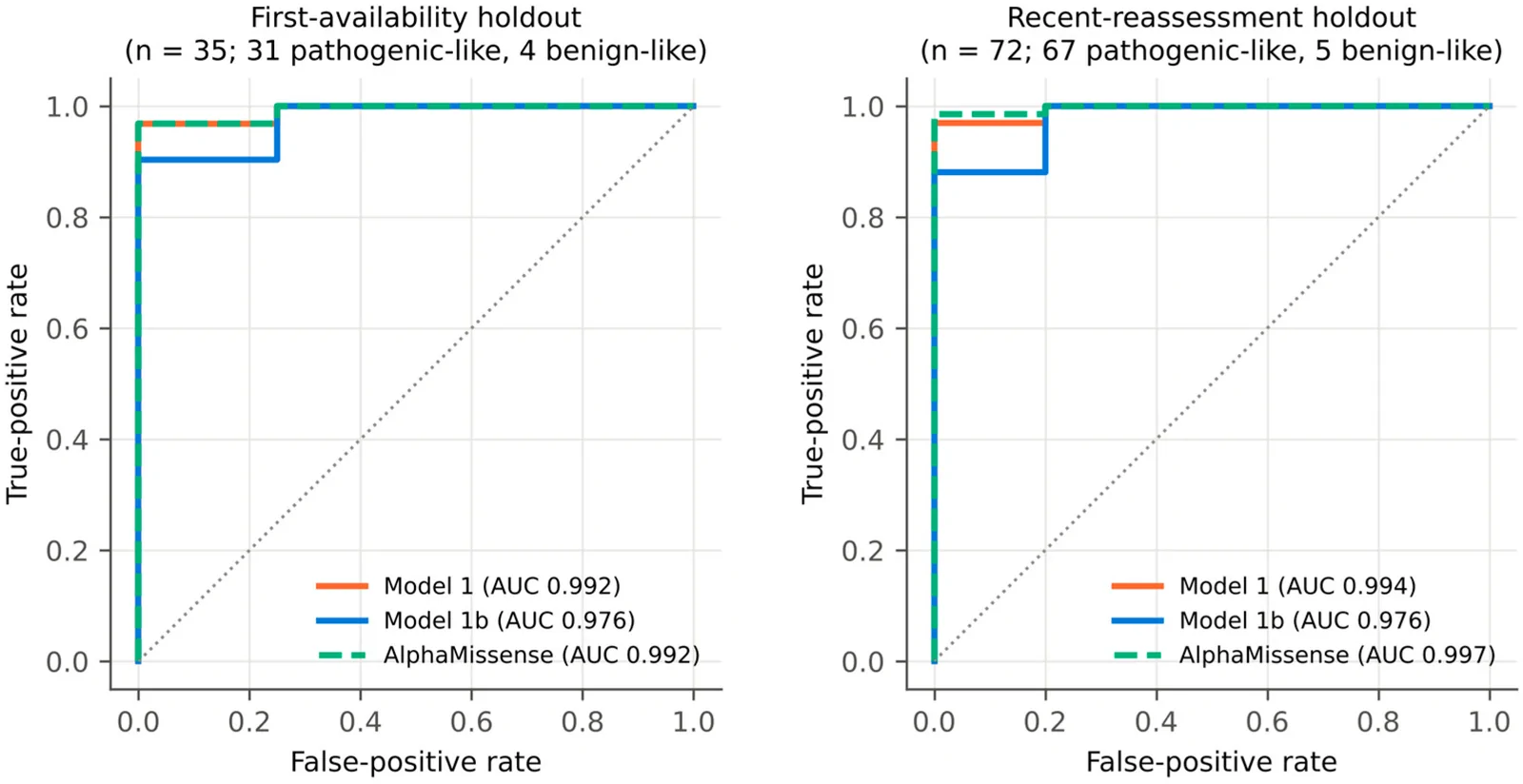
ROC curves for Model 1 (full evidence), Model 1b (structural/conservation), and AlphaMissense on the first-availability holdout (**left**) and the recent-reassessment holdout (**right**).

External validation used two temporal splits (Table 2 and Table S5, Figure 2). In the recent-reassessment holdout, each variant is dated by the year its classification was last evaluated, so some test variants had been published long before 2025 (for example, C36Y and R67W, first reported in 2006). The stricter first-availability holdout therefore dates each variant by the earlier of its first ClinVar evaluation and its first literature report. Variants first available before 2025 formed the training partition (178 variants, seven benign-like) and those first available in 2025–2026 formed the test partition (35: 31 pathogenic-like, four benign-like). On this holdout, Model 1b scored 0.976 (95% CI 0.896–1.000) and Model 1 0.992 (0.958–1.000), versus 0.992 for AlphaMissense. On the recent-reassessment holdout (72 variants, five benign-like), the values were 0.976, 0.994, and 0.997. Paired DeLong tests found no significant difference from AlphaMissense in any comparison (*p* = 0.35–1.00). Because a non-significant DeLong test does not establish equivalence, I also ran a two one-sided-tests (TOST) analysis with a pre-specified ±0.05 AUC margin. Equivalence was supported for Model 1 on both holdouts (TOST *p* = 0.002 and <0.001) but not for Model 1b (TOST *p* = 0.07 and 0.10). With four or five benign test variants, the holdouts cannot exclude a gap of several AUC points for Model 1b. Both holdouts therefore give preliminary evidence of generalizability, not confirmation of performance in an independent clinical dataset. Ninety-eight labeled variants without any date could not be placed in either partition.

### 2.3. Sensitivity to Label Quality

ClinVar classifications can themselves draw on REVEL or CADD (ACMG PP3/BP4), which raises a circularity concern for Model 1. REVEL does not take allele frequency as an input. It was, however, trained to separate HGMD disease mutations from rare population variants, so its scores may partly reflect the same literature on which the pathogenic labels rest. Model 1 also uses gnomAD allele frequency directly. Model 1b uses neither REVEL nor allele frequency and is free of both sources. I therefore refit both models on a stricter high-confidence subset of the 311 labels: variants with a ClinVar review status of two stars or more, or with a concordant published functional-assay result. A single normal assay result does not establish clinical benignity; R200G and D176Y, for example, are pathogenic in ClinVar. Assay results were therefore used only to confirm a label, never to create a benign one (Section 4.5). The resulting subset has 117 variants (108 pathogenic-like, nine benign-like). Model 1 scored 0.995 (95% CI 0.981–1.000), Model 1b 0.930 (0.859–0.988), and AlphaMissense 0.974 on this subset (Table S6). Restricting to the best-supported labels therefore changed Model 1b by about two AUC points and did not lower Model 1.

Across 20 random seeds on this subset, Model 1 scored 0.994 ± 0.002 and Model 1b scored 0.920 ± 0.014. With only nine benign-like variants, these estimates carry wide confidence intervals.

### 2.4. Population-Frequency Expansion of the Benign Class (Sensitivity Analysis)

The benign class is small (15 of 311 labels). A gnomAD allele-frequency threshold of 1 × 10^−5^ would add 50 VUS-like variants to the benign class. This threshold is lower, and therefore less conservative for benign assignment, than the disease-specific maximum credible allele frequency derived with the standard formula [17] (≈1.4 × 10^−5^; Section 4.8). It is also far below the thresholds of the ClinGen HHT Variant Curation Expert Panel, which assigns BS1_Supporting above a Popmax/Grpmax filtering allele frequency of 0.08% and BS1 above 0.2% [18]. No VUS-like variant in the dataset reaches either threshold. An audit of the 50 added variants found 14 with contrary evidence, including H66Y (defective in a functional assay and reported as pathogenic) and R200W (conflicting ClinVar classifications; AlphaMissense 0.94) (Table S7). The expansion is therefore reported only as a sensitivity analysis.

Adding population-frequency-defined benign variants did not improve Model 1b at any threshold (Table 3). With gnomAD allele frequency removed from Model 1 to avoid circularity, Model 1b scored 0.947 ± 0.008 without expansion. It scored 0.937 ± 0.006 with the 50 variants added at 1 × 10^−5^, 0.937 ± 0.006 with 43 added at 1.4 × 10^−5^, and 0.917 ± 0.003 with five added at 8 × 10^−5^. Model 1 changed by less than 0.01 in either direction. At the expert-panel thresholds, no variant would be added, so the models are unchanged.

### 2.5. PAH Versus HHT Phenotype (Exploratory)

**Figure 3:**
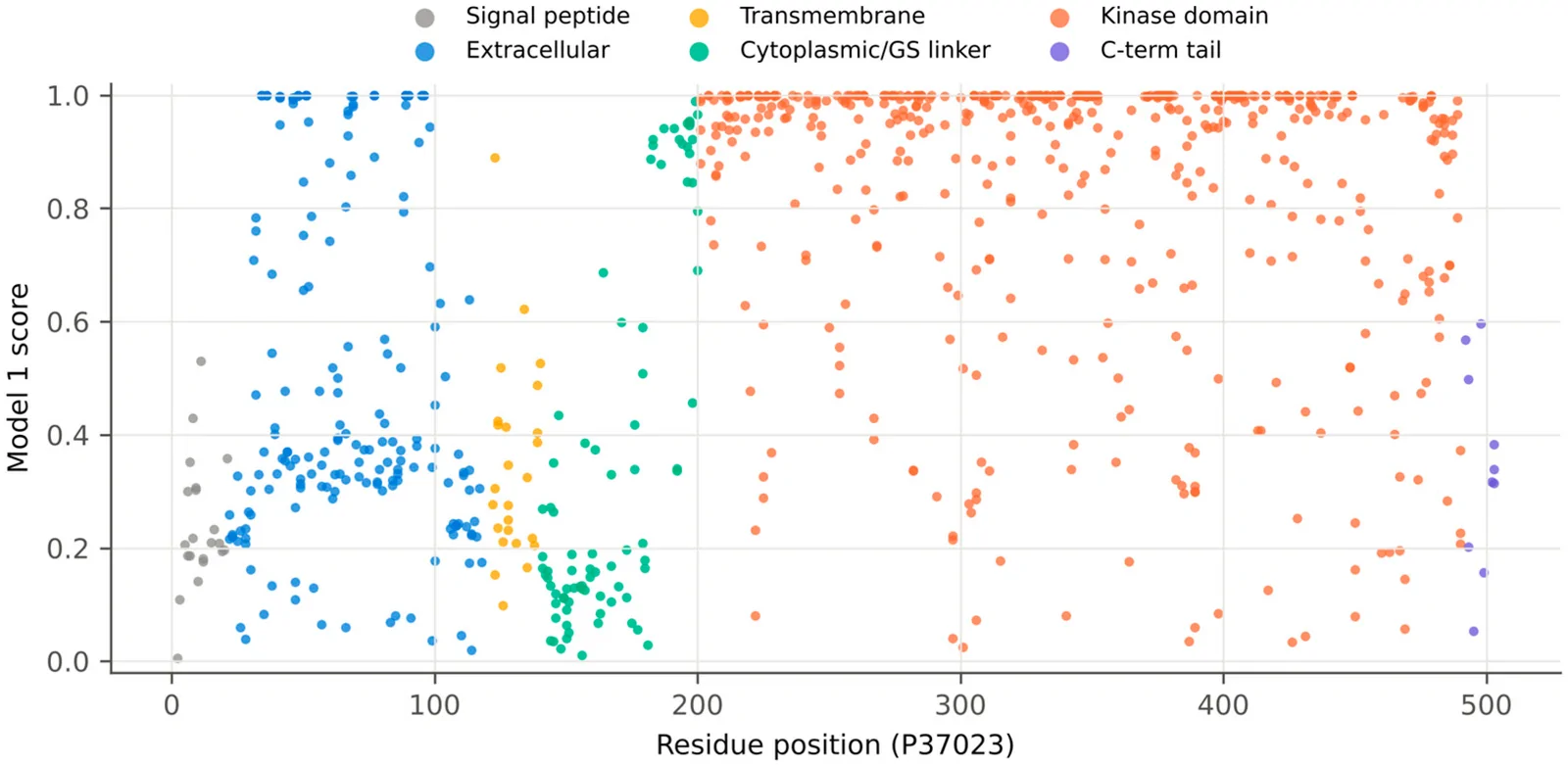
Model 1 pathogenicity score by residue position, colored by domain, for the 886-variant screen (out-of-fold scores for training variants). The 13 post hoc additions, which have sparser feature coverage, are excluded.

As an exploratory analysis, a random-forest classifier was trained to separate variants reported in PAH from those reported in HHT, using only structural, conservation, and positional features. Phenotype tags came from the PubTator3/LitVar2 annotation of the citing publications and were verified against the variant-level text of each cited publication for every variant with a PAH tag. On 112 variants with a single phenotype tag (98 HHT, 14 PAH; 8 with both tags were excluded), the model reached a cross-validated ROC-AUC of 0.72 (95% CI 0.57–0.84). The 20-seed mean was 0.672 ± 0.039, and the PR-AUC was 0.28 against a baseline of 0.125. To test the kinase-domain enrichment reported by Girerd et al. [2], the proportion of PAH-tagged variants was compared between the two large domains. Of the 112 variants, 80 lie in the kinase domain (11 PAH-tagged, 13.8%) and 26 in the extracellular domain (two PAH-tagged, 7.7%). The remaining six (five in the GS linker, none PAH-tagged, and one PAH-tagged signal-peptide variant) were too few to compare. The difference is in the direction reported by Girerd et al. but not significant (Fisher’s exact *p* = 0.51). With 14 PAH variants, this analysis is underpowered, and the model is not a validated phenotype classifier. Figure 3 shows Model 1 scores by residue position.

### 2.6. Structural Quality Control

**Figure 4:**
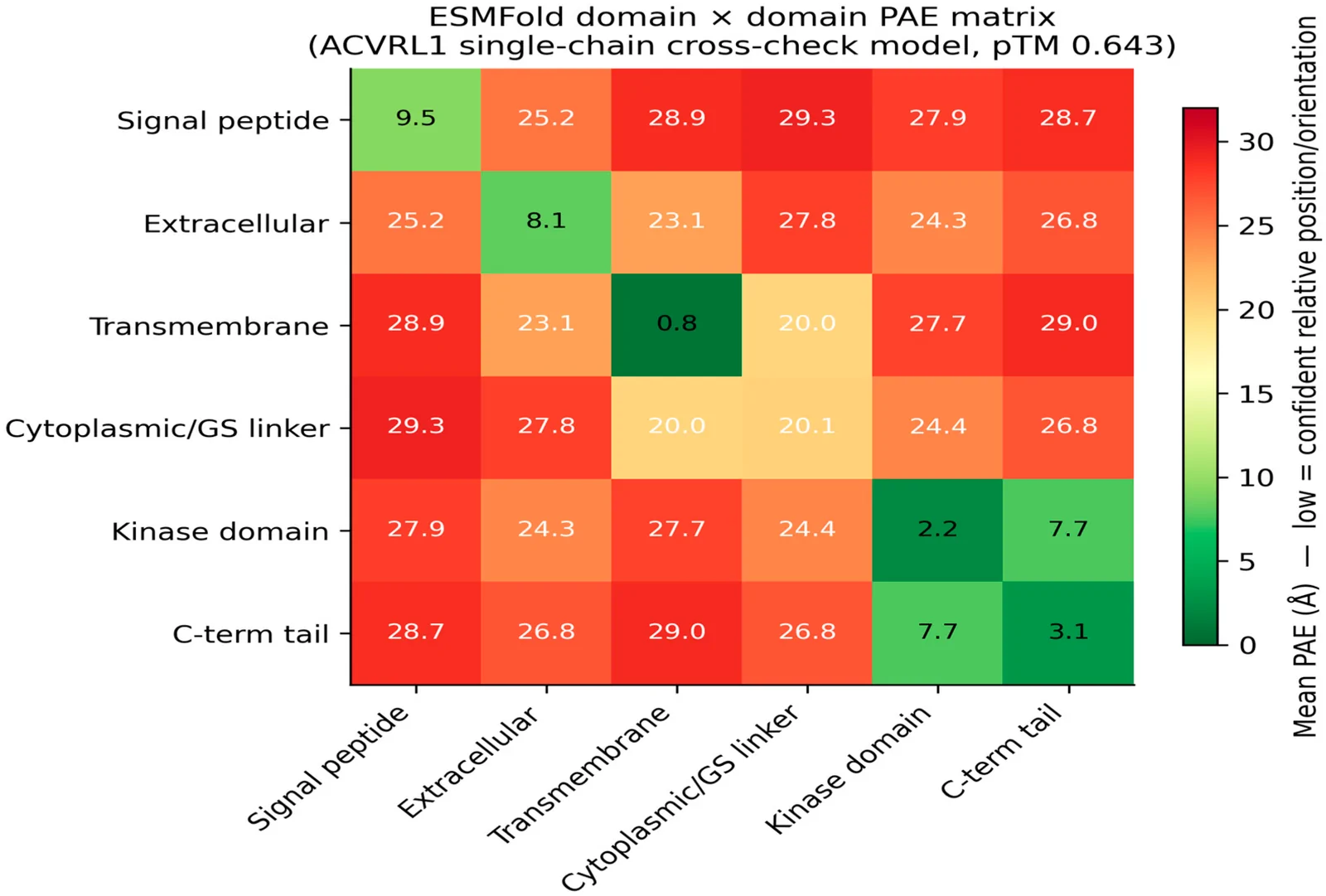
Domain × domain mean predicted aligned error (PAE) matrix for the independent ESMFold cross-check structure (Section 2.6), summarizing confidence in inter-domain relative orientation. A matching PAE matrix was not available for the AlphaFold3 structure used elsewhere in this study.

FoldX and DynaMut2 ΔΔG calculations depend on having a trustworthy structural model. I cross-checked the AlphaFold3 monomer model used for FoldX and the other single-variant structural features (AlphaFold Server; ranking_score 0.78, pTM 0.67) against an independently generated ESMFold [19] structure (pTM 0.643). ESMFold is a protein-language-model predictor that uses no multiple sequence alignment. It is therefore mechanistically distinct from AlphaFold’s MSA-based Evoformer, so agreement between the two is a stronger check than comparing two MSA-based tools. Per-domain pLDDT agreement was generally good. The largest disagreement (28.25 pLDDT points) was in the cytoplasmic GS-linker region, which contains the R200G worked example below; that example is therefore presented with this caveat. A predicted-aligned-error (PAE) domain-orientation-confidence analysis, available only for the ESMFold structure, is summarized in Figure 4 and Table S8. The overall confidence of this ESMFold model (pTM 0.643) is modest by conventional standards, under which structures are usually considered well resolved above about 0.7–0.8. The AlphaFold3 model’s own pTM (0.67) is comparable. Figure 4 shows why: inter-domain mean PAE is high (about 25–29 Å) for almost every domain pair off the diagonal. The ESMFold model therefore has limited confidence in the relative arrangement of its domains throughout the chain, not just at the GS linker. Within-domain confidence is, by contrast, good (intra-domain mean PAE 2–9 Å for the extracellular, kinase, and C-terminal domains; higher, ≈20 Å, for the GS-linker itself, consistent with the pLDDT disagreement above). The single-variant ΔΔG and conservation features in this study come from the AlphaFold3 structure, not from ESMFold, and they depend on local fold geometry rather than inter-domain orientation. This PAE limitation therefore applies most directly to the cross-check structure itself. Both models, however, share the same regions of low pLDDT agreement, so similarly modest inter-domain confidence in the AlphaFold3 model is a reasonable expectation in those regions, though not a demonstrated one. This remains a disclosed uncertainty that the cross-check does not resolve.

Two further checks were added. First, the AlphaFold3 monomer places the extracellular domain against the kinase domain (15 inter-domain residue contacts under 4 Å). These domains lie on opposite sides of the membrane, so this interface is an artifact of predicting a single-pass receptor as a soluble monomer; only within-domain geometry from this model is used. Second, the AlphaFold Database model used for the exploratory molecular-dynamics screen agrees with the AlphaFold3 monomer within each folded domain but not in domain arrangement (global RMSD 26.6 Å; Table S9). Within domains, the Cα RMSD is 0.29 Å for the extracellular domain, 0.21 Å for the transmembrane helix, and 1.16 Å for the kinase domain, over residues with pLDDT ≥ 70 in both.

**Figure 5:**
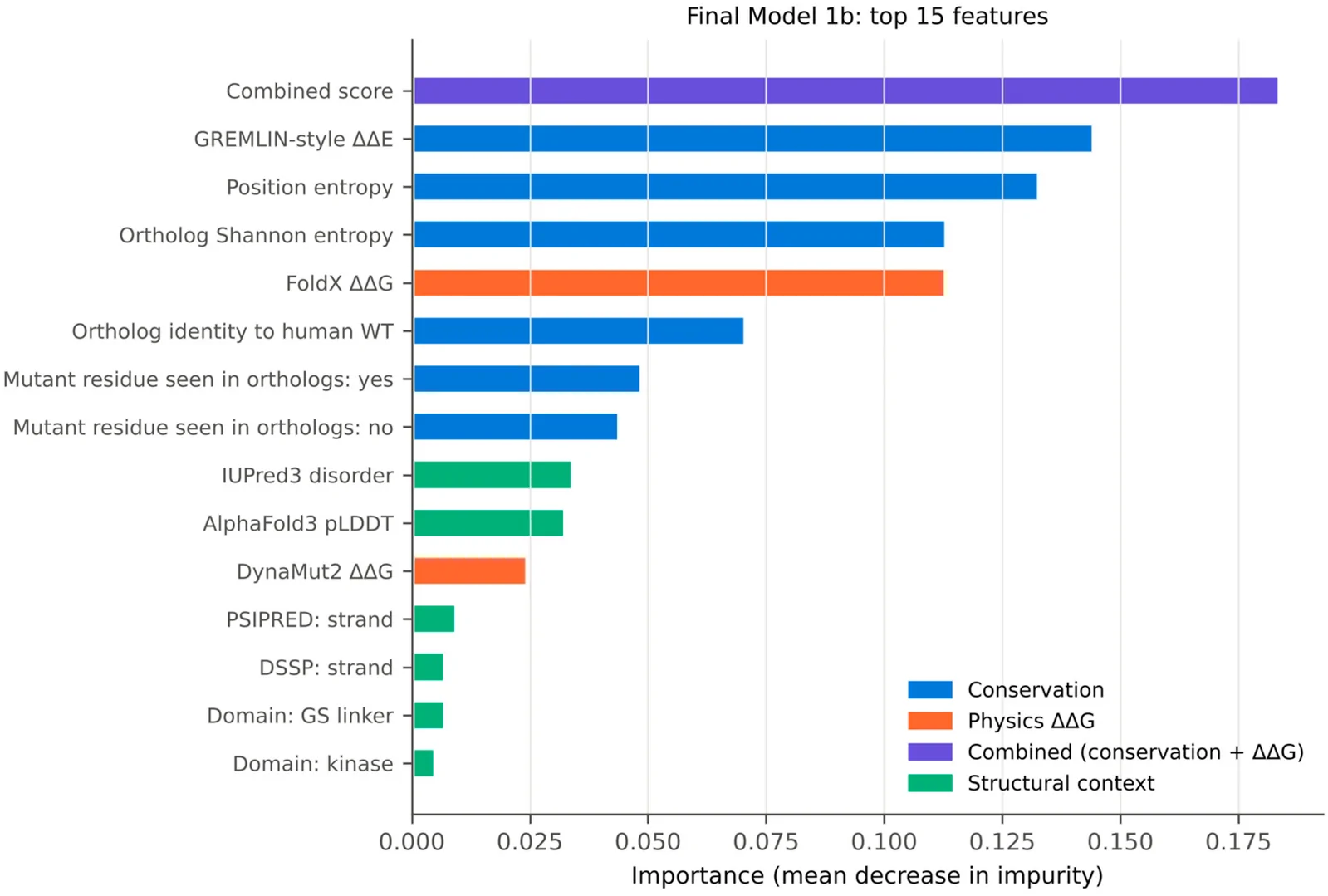
Random-forest feature importances for the final Model 1b, top 15 features, colored by evidence type. Combined_score is the sum of z-scored GREMLIN-style ΔΔE and z-scored FoldX ΔΔG and is shared between the conservation and physics categories.

Feature-group ablations and per-variant attributions were used to test which evidence drives Model 1b (Figure 5, Tables S4 and S10). Conservation features alone reached a cross-validated ROC-AUC of 0.929 ± 0.007, ΔΔG features alone reached 0.817 ± 0.026, and Model 1b without conservation features reached 0.830 ± 0.025. On their own, the molecular-dynamics features reached an ROC-AUC of 0.643 ± 0.029 and the electrostatic features reached 0.396 ± 0.015; the latter is below chance and carries no usable signal. Adding both groups did not improve either model (seed-42 ROC-AUC with and without them: Model 1b 0.949 and 0.954, Model 1 0.980 and 0.979), so neither group was used. Out-of-fold SHAP attributions, summed by evidence group, identified conservation as the dominant group for 274 of 311 variants (88%), the combined conservation/ΔΔG score for 13, ΔΔG for 19, and structural context for five. Evolutionary constraint therefore drives most calls, and predicted destabilization contributes to a minority. The attribution shows which type of evidence a call rests on; it does not by itself establish a biological mechanism. A conservation-driven call identifies a constrained residue and is a biologically informed hypothesis that the substitution impairs ALK1, not direct evidence of functional impairment.

### 2.7. Worked Examples: The Evidence Behind Individual Calls

**Figure 6:**
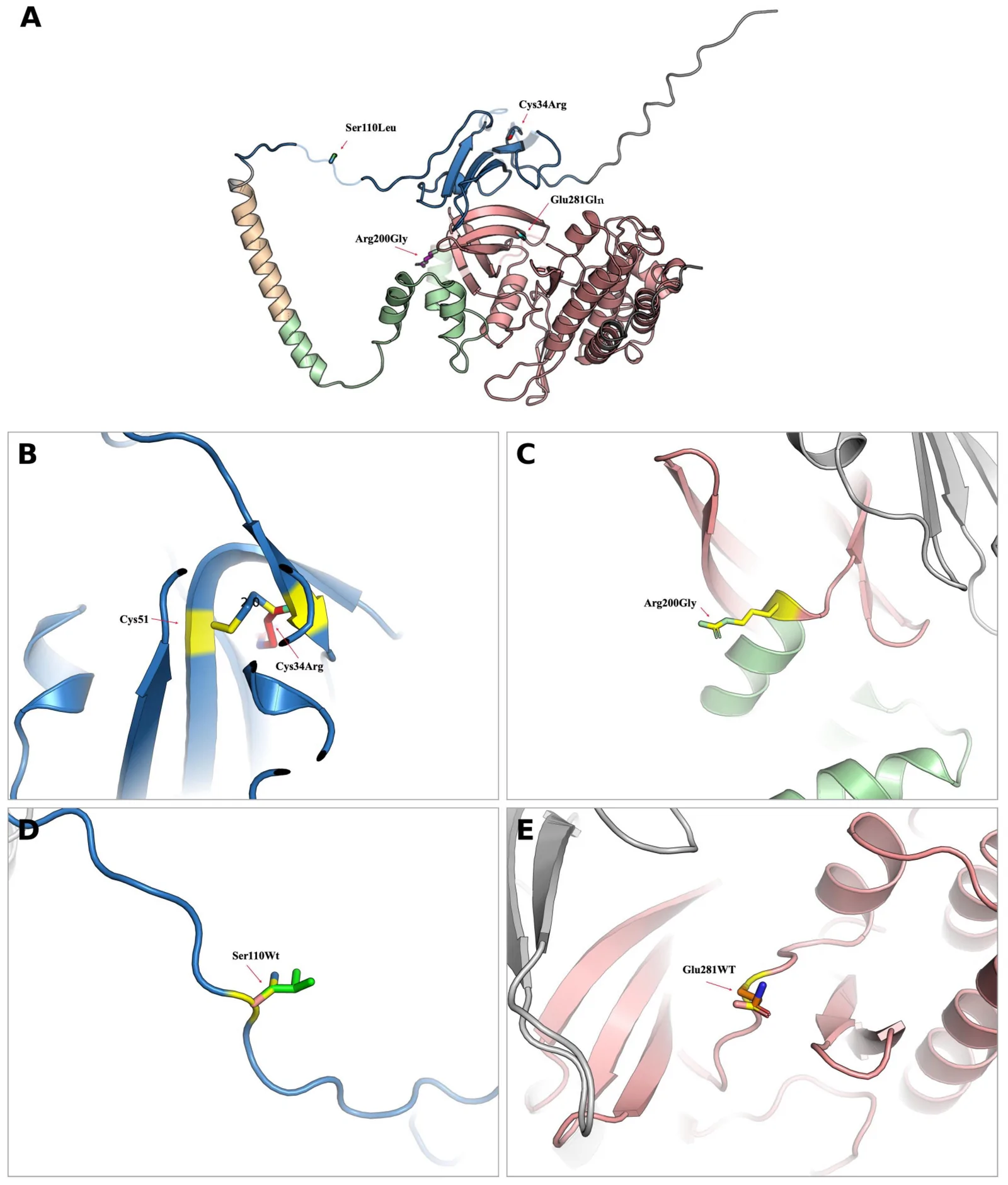
PyMOL cartoon-and-stick renderings of the four worked-example residues (Section 2.7) on the project’s AlphaFold3 monomer model of full-length ACVRL1, generated by the accompanying script (worked_examples_pymol.pml, Supplementary Material; Section 4.9). Panel (**A**) shows all four positions together on the domain-colored whole chain. Panels (**B**–**E**) show each variant with its wild-type and in-place modeled mutant side chains, for illustration only; locally relaxed mutant models are provided as File S1.

Four variants illustrate how the per-variant evidence can be read (Figure 6). Scores are out-of-fold for training variants and out-of-sample for E281Q. The mechanistic readings below are hypotheses unless supported by functional data.

C34R (extracellular domain): FoldX ΔΔG is +26.1 kcal/mol, among the most destabilizing values in the screen, and Cys34 is nearly invariant (96.4% ortholog identity; GREMLIN-style ΔΔE at the feature’s ceiling). In the AlphaFold3 model, Cys34 forms a disulfide bond with Cys51 (Sγ–Sγ 2.00 Å), so the substitution is expected to remove this bond. FoldX does not model loss of a covalent disulfide, and +26.1 kcal/mol lies far outside its validated range, so only the direction of this estimate should be trusted. In the unrelaxed mutant model, Arg34 clashed with neighboring atoms (27 heavy-atom contacts under 3.0 Å, closest 2.0 Å); local energy minimization removed all of them. It is pathogenic in ClinVar, and the scores are 1.00 for Model 1b, 1.00 for Model 1 and 0.984 for AlphaMissense.

R200G (GS linker/kinase-domain boundary): FoldX ΔΔG is near neutral (+0.31 kcal/mol) in a region with low structural confidence (Section 2.6), whereas conservation is high (87.3% ortholog identity). It is pathogenic in ClinVar (single submitter), and the reported phenotype is HHT. The scores are 0.96 for Model 1b, 0.97 for Model 1 and 0.971 for AlphaMissense. One functional assay found normal activity for R200G; because a single normal assay result does not establish benignity, it keeps its ClinVar pathogenic label (Section 2.3). The example shows that conservation can remain informative where the structural model is unreliable. The call marks a constrained residue but does not show how R200G would impair ALK1, and the normal assay result means that this remains a hypothesis to test.

S110L (extracellular domain, benign): FoldX ΔΔG is −0.51 kcal/mol, and the position is poorly conserved (24.3% ortholog identity; Shannon entropy 3.49 of a possible 4.39 bits). It is benign/likely benign in ClinVar, and the scores are 0.12 for Model 1b, 0.05 for Model 1 and 0.064 for AlphaMissense. The classifier therefore does not call every unusual substitution pathogenic.

E281Q (kinase domain, VUS): E281Q has been reported once, as a novel variant predicted to be pathogenic, without segregation or functional data [20]. It is therefore classified here as a VUS and was excluded from training. Scored out of sample, Model 1b assigns 0.97 and Model 1 0.96, driven mainly by conservation (94.5% ortholog identity; GREMLIN-style ΔΔE at ceiling), whereas AlphaMissense assigns 0.145. FoldX predicts no destabilization (+0.43 kcal/mol), and the Glu281 side chain is solvent-exposed in the AlphaFold3 model (112 Å^2^ accessible surface area). The discordance therefore rests on evolutionary constraint rather than on a structural mechanism, and the pathogenic-range call is a hypothesis rather than evidence of impaired function. A BMP9–SMAD reporter assay would resolve it. BMP9 signals through ALK1 to activate SMAD1/5/9, so the assay measures ALK1 function directly. It would show whether E281Q impairs signaling despite its predicted stable fold, which is the point on which the conservation-based models and AlphaMissense disagree.

(A) Whole-chain, domain-colored overview with all four variant positions marked (signal peptide, gray; extracellular domain, blue; transmembrane helix, tan; cytoplasmic/GS linker, green; kinase domain, salmon; C-terminal tail, dark gray).

(B) C34R, extracellular domain, wild-type Cys34 side chain (yellow) and PDBFixer-modeled mutant Arg34 side chain (red), with the computationally confirmed Cys34–Cys51 disulfide bond (Sγ–Sγ = 2.00 Å) indicated.

(C) R200G, GS linker/kinase-domain boundary, wild-type Arg200 (yellow) and mutant Gly200 (orange).

(D) S110L, extracellular domain, correctly called benign; wild-type Ser110 (yellow) and mutant Leu110 (green).

(E) E281Q, kinase domain, wild-type Glu281 (yellow) and mutant Gln281 (orange); the side chain is solvent-exposed.

In each case the score comes with the evidence that drives it, checked against independent data where they exist (disulfide geometry, ClinVar status and, for R200G, a functional assay). Where no such data exist (E281Q), the attribution points to a specific experiment.

### 2.8. Comparison to a Zero-Shot Genomic Foundation Model (Evo2)

As an independent check unrelated to any ACVRL1-specific training, I scored the 565 variants with sufficient genomic-coordinate resolution (Section 4.11) using Evo2 [21]. Evo2 is a 7-billion-parameter genomic foundation model trained on 9.3 trillion nucleotides spanning all domains of life; it was accessed via NVIDIA’s hosted inference API, with no ACVRL1- or HHT-specific fine-tuning. For each variant, Evo2 scored an 8192 bp reference window and the corresponding variant window carrying the single-nucleotide substitution. The delta log-likelihood (more negative = more disruptive to the model’s learned sequence expectations) was computed as in the model’s own published zero-shot variant-effect protocol.

**Figure 7:**
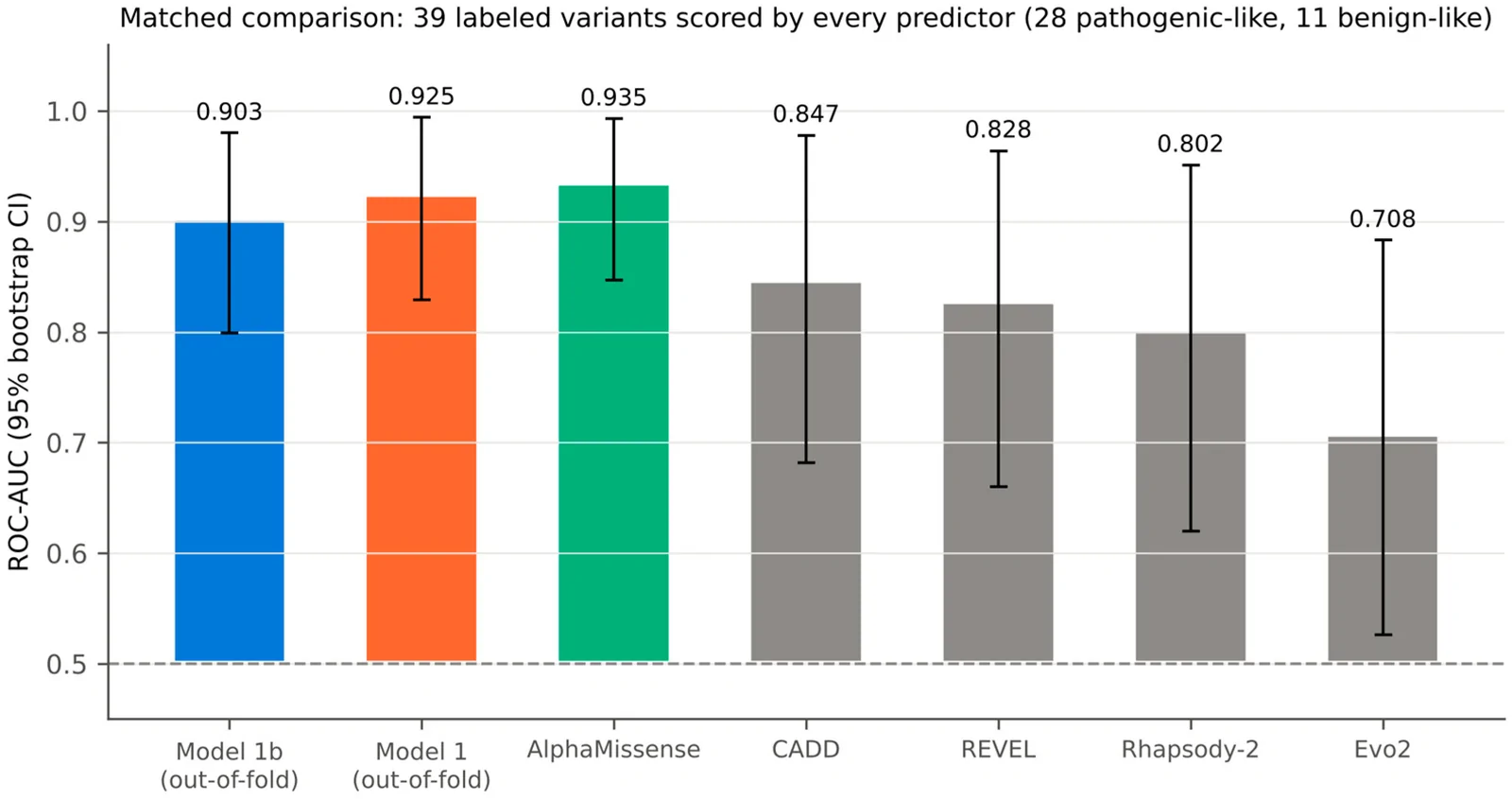
ROC-AUC (95% bootstrap CI) of Model 1b and Model 1 (out-of-fold), AlphaMissense, CADD, REVEL, Rhapsody-2 and Evo2 on the same 39 labeled variants scored by all predictors (28 pathogenic-like, 11 benign-like). Dashed line = chance.

Of the 311 labeled variants, 309 have an Evo2 score (13 benign-like), and Evo2’s delta reached an ROC-AUC of 0.797 (95% CI 0.656–0.921). REVEL and CADD cover far fewer labeled variants, so all predictors were compared on the 39 variants scored by REVEL, CADD, and Evo2 (28 pathogenic-like, 11 benign-like). Models 1b and 1 were represented by their out-of-fold predictions (Figure 7). Out-of-fold predictions are not circular, so the classifiers can be compared with all other predictors on identical variants. On this matched set, ROC-AUCs were 0.903 for Model 1b, 0.925 for Model 1, 0.935 for AlphaMissense, 0.847 for CADD, 0.828 for REVEL, 0.802 for Rhapsody-2, and 0.708 for Evo2. Across all 565 scored variants, Evo2’s delta correlated moderately with the other predictors (|Spearman ρ| = 0.38–0.59, all *p* < 10^−6^).

Evo2 uses no protein structure and no ACVRL1-specific training, and returns a single likelihood-based score. It performs well above chance but falls short of AlphaMissense and of the gene-specific classifiers on the same variants. The matched set has only 11 benign-like variants, so the differences among the leading predictors are uncertain.

### 2.9. Benchmark Against Rhapsody-2, an Independent Mechanistic Predictor

Banerjee et al.’s Rhapsody-2 [15] independently combines short-timescale molecular dynamics, structure, and true pairwise evolutionary coevolution into a single pretrained pathogenicity score, offering a natural, non-ACVRL1-specific comparator to this study’s own physics-and-conservation classifier. Rhapsody-2’s own published code (github.com/anupam-banerjee/rhapsody-2, not the older, unrelated 2020 ‘prody-rhapsody’ PyPI package) was cloned and run locally for all 886 variants in the original screen (Section 4.10). It used the same ESMFold ACVRL1 structure as the independent structural cross-check in Section 2.6. Its evolutionary-conservation branch requires a live NCBI BLAST search. A BLASTP query (database clustered_nr, E-value threshold 0.001, query P37023) was therefore submitted directly to NCBI, and its results were supplied to Rhapsody-2 in place of its own timeout-limited automated call. This allowed the complete feature set (dynamics, structure and evolutionary conservation) to be run.

Rhapsody-2 ships a labeled database (rhapsody2_sav_db.tsv, 117,525 variants across about 12,000 proteins) that is likely part of its training data. All 165 ACVRL1 entries in it are in this study’s screen and were excluded from the independent-subset analysis. On the 311 labeled variants, Rhapsody-2’s full-feature score reached an ROC-AUC of 0.904 (*n* = 311). On the independent subset it reached 0.997 (*n* = 157, seven benign-like), where Model 1b scored 0.965, Model 1 0.969, and AlphaMissense 0.995 on the same variants. Rhapsody-2 therefore ranked the independent labeled variants as well as AlphaMissense and better than Model 1b, although with seven benign-like variants these differences are uncertain. Per-variant scores are in Table S11.

## 3. Discussion

This study does not show that an interpretable classifier outperforms AlphaMissense for ACVRL1. It shows that a classifier built from conservation and stability estimates, without population-frequency data, reaches a cross-validated ROC-AUC of 0.954 on documented labels. It also performs close to AlphaMissense on a first-availability holdout, which, with four benign test variants, is preliminary evidence of generalizability. Formal equivalence within ±0.05 AUC was supported only for Model 1, which also uses sequence-based predictors; for Model 1b, the holdouts are too small to establish or exclude equivalence. Benign recognition is the main weakness. Model 1b correctly classified 10/15 benign variants against 14/15 for AlphaMissense, so its high ROC-AUC hides a material benign error rate. The added value of the approach is the per-variant attribution, which a black-box score cannot provide. AlphaMissense scores C34R and S110L correctly, but only the structure-based reading shows that C34R is expected to break the Cys34–Cys51 disulfide bond and that S110L lies at a poorly conserved position with no predicted destabilization. For most variants, the call rests on evolutionary constraint, and for a minority, predicted destabilization contributes. The attribution therefore tells a laboratory what kind of evidence supports a call and which experiment would test it. It is not functional evidence in the ACMG PS3/BS3 sense, and each mechanistic reading remains a hypothesis until tested. The worked examples are consistent with ClinVar status and, where available, functional data, and E281Q shows how a discordant call can be turned into a specific experiment.

Table 4 compares the two classifiers with established predictors. For a patient with suspected HHT, PAH, or both who carries an ACVRL1 missense VUS, the score could contribute computational evidence under the ACMG/AMP PP3 criterion. ACMG/AMP counts computational evidence only once, so the score would replace, not add to, REVEL or AlphaMissense. ClinGen recommends that the evidence strength of each tool be calibrated against independent labeled variants before use [22]. Until such a calibration exists, the score can provide at most supporting evidence and should not be used for BP4. False-positive calls are the main clinical risk. At the 0.5 threshold, Model 1b scored five of the 15 benign-like variants as pathogenic. Four of them lie at confidently modeled kinase-domain residues, and three (R218W, L220F, and A482V) meet an HHT VCEP population-frequency code [18]. A pathogenic-range score must therefore be read together with allele frequency, which Model 1b deliberately does not use. What the score adds to other computational tools is the attribution. It shows whether a call rests on conservation or on predicted destabilization, which can guide the choice of functional assay (BMP9–SMAD reporter or cell-surface expression). Because Model 1b scored one in three benign-like variants as pathogenic, a pathogenic-range score cannot by itself distinguish a benign VUS from a pathogenic one. According to the ACMG/AMP framework and ClinGen recommendations, computational predictors can contribute evidence at different strengths (supporting, moderate, or strong), depending on their calibration and validated performance. A predictor alone is insufficient to determine a variant’s classification. However, its results can contribute to classification when integrated with other available evidence, including the gene-specific disease mechanism and the relevant clinical, genetic, population, and functional evidence. The most realistic near-term use of Model 1b is to prioritize VUS for functional testing or family segregation studies, not to determine pathogenicity independently. A resolved classification allows predictive testing of relatives and targeted screening for pulmonary, cerebral, and hepatic arteriovenous malformations or for PAH. The classifier has not been calibrated or validated for clinical use, and better interpretability is not the same as demonstrated clinical utility.

Structural uncertainty affects individual calls unevenly. Of the 311 labeled variants, 294 lie at residues with AlphaFold3 pLDDT ≥ 70 and 17 below. Benign-like variants are concentrated in the low-confidence regions (7 of 17, against 8 of 294), mainly the GS linker and flexible parts of the extracellular domain. Because pLDDT is itself a feature, part of its signal reflects where a variant lies rather than a mechanism. Structural uncertainty does not explain the false-positive calls, because four of the five lie at residues with pLDDT 87–97. A simple confidence rule can guide interpretation. Where the residue pLDDT is below 70, or the AlphaFold3 and ESMFold models differ by more than 20 pLDDT points, the ΔΔG contribution should not be read as a mechanism. The call then rests on conservation alone, as for R200G (Section 2.7). Both confidence values are given for every variant in Table S1.

The two-hit model of HHT [4,5,7] clarifies the role of germline classification. A pathogenic germline ACVRL1 variant identifies who carries the necessary first hit and is the basis for diagnosis, cascade testing, and counseling. It does not predict the number, location, or timing of malformations, which depend on somatic second hits. Germline classifiers, including this one, address only the first question.

Limitations: First, this is a single-gene study, and its thresholds, feature weights, and conservation alignment do not transfer to other genes without re-derivation. Second, the benign class is small (15 of 311 labels), benign recall estimates have wide confidence intervals, and population-frequency expansion did not resolve this (Section 2.4). No predictor was used to select or label benign variants: 14 are ClinVar benign or likely benign, and one (Q150R) exceeds the BA1 population-frequency threshold (Table S3). Indirect circularity remains possible, because ClinVar submitters may cite in silico evidence (BP4) and AlphaMissense was trained with common population variants as benign. However, restricting the benign class to the five expert-panel classifications did not favor AlphaMissense (ROC-AUC 0.964, versus 0.982 for Model 1 and 0.925 for Model 1b). Third, the temporal holdouts contain only four or five benign variants. They give preliminary evidence of generalizability but cannot establish equivalence for Model 1b, and confirmation needs an independent clinical cohort with more benign variants. Fourth, 98 labeled variants had no date and could not be placed in either temporal partition. Fifth, the AlphaFold3 monomer places the extracellular domain against the kinase domain, an artifact of monomer prediction, so only within-domain geometry is used. Sixth, the PAH/HHT analysis (Section 2.5) rests on 14 PAH variants and is exploratory. Seventh, 165 of the 886 screened variants are probably in Rhapsody-2’s training data, so the independent-subset estimates are the more defensible ones. Finally, the 13 post hoc literature-mined variants have sparser feature coverage, and their scores should be read with caution.

Future directions: The most direct next step is a BMP9–SMAD reporter assay for E281Q, which would test a call driven by conservation alone. Longer, explicit-solvent, membrane-embedded simulations of selected variants could test whether dynamics add information that the short implicit-solvent screen could not provide. A larger, family-wide alignment would allow a true pairwise coevolution term (Section 4.3).

## 4. Materials and Methods

### 4.1. Variant Assembly

In total, 899 ACVRL1 missense variants (886 from the original structural/conservation screen plus 13 post hoc additions) were curated from ClinVar [23], gnomAD v4.1.1 [24], dbSNP, and the literature, using PubTator3/LitVar2 full-text mining [25]. Individual published case reports were identified with the help of HGMD Professional (release 2026.3) and PubTator3; each variant is cited by its original publication, and no HGMD classifications or accession numbers are redistributed. The 13 additions comprise 12 variants from a full-text PubTator3 sweep and one individually cited case report (A347G); they have sparser feature coverage and are flagged wherever they are scored. Training labels and their sources are listed in Table S3 (Section 2.1).

### 4.2. Structural and Physics-Based Features

FoldX 5.1 [26] ΔΔG was computed on the AlphaFold3 monomer (RepairPDB, then BuildModel); positive values are destabilizing. DynaMut2 [27] ΔΔG was computed on experimental structures: PDB 6SF3 chain A (ALK1 extracellular domain, residues 30–105, from the BMP10 complex) and PDB 3MY0 chain A (kinase domain, residues 195–493). Negative values are destabilizing, and the values were used without sign change. DynaMut2 covered 722 of 886 variants, and each uncovered variant has a documented reason. The structure used for each feature is listed in Table 5. Secondary structure and solvent accessibility used PSIPRED 4.0 (UCL PSIPRED web server) [28] and DSSP 4.4.11 (Phenix 2.0) [29], intrinsic disorder IUPred3 [30], and interface membership (BMPR2, FKBP1A) a PISA-style interface analysis following the PDBePISA definitions [31], with Shrake–Rupley surface areas computed in Biopython. An exploratory molecular-dynamics screen (OpenMM 8.5.1 [32]; AMBER14 with GBSA-OBC2 implicit solvent, minimization plus 50 ps at 300 K) was run on the AlphaFold Database model, and per-variant electrostatic descriptors were also computed. These trajectories are not converged, and an ablation showed that neither feature group added predictive value (Table S4), so neither was used in the classifiers. For the worked examples, the four mutant models were locally relaxed with OpenMM 8.6.1 (AMBER14/OBC2; atoms within 10 Å of the mutated residue were mobile, the rest being fixed; energy minimization to convergence).

### 4.3. GREMLIN-Style Conservation Score

A field-only (single-site) conservation score, regularized in the style of GREMLIN [33], was computed from a multiple sequence alignment of unique ACVRL1 orthologs. Orthologs were retrieved from UniProt (query gene: ACVRL1 AND length: [400 TO 550], reviewed and unreviewed) and deduplicated to one sequence per species. They were aligned with FAMSA 0.7.0 using a UPGMA guide tree, and the alignment was trimmed to the non-gap match columns of human P37023. Sequences were reweighted at 80% identity clustering (weight = 1/cluster size). The effective sample size Neff was 2.43 for the original 53-ortholog alignment and 5.07 for a rebuilt 45-ortholog alignment used for later-added variants. The rebuilt alignment was re-fetched and realigned independently because the original was not preserved; it uses the identical field-term formula. The field term is freq = (counts + λ/q)/(Neff + λ), λ = 1.0, q = 21 (20 amino acids + gap); V_i = log(freq); ΔΔE = log(f_WT) − log(f_mut). Pairwise coupling terms were attempted, not skipped by default. Neff/L was ≈0.005 for the ACVRL1-only alignment, and Neff reached only ≈9.7 after expanding to a 6-paralog family alignment (ACVR1, ACVR1B, ACVR1C, BMPR1A, BMPR1B, TGFBR1, and their orthologs; 194 sequences). Both were judged statistically insufficient for reliable estimation of pairwise couplings. This is therefore a single-site, field-only conservation score, not a pairwise-coevolution score, and is described as such throughout, including in comparisons against genuinely coevolution-based tools (Rhapsody-2). The separate ortholog conservation statistics used as additional Model 1b features were percent identity to the human wild-type residue, Shannon entropy, and whether the mutant residue is observed in any ortholog. They were derived from an independent phmmer (HMMER 3.3.2 [34]; run via the ConSurf web server) homology search against the same ortholog set, not recomputed from the GREMLIN-style alignment.

### 4.4. Model 1/Model 1b: HHT Pathogenicity Classifiers

Both classifiers are random forests (scikit-learn [35]; 500 trees, maximum depth 6, min_samples_leaf 2, class_weight = ‘balanced’). Numeric features were median-imputed and standardized, and categorical features were one-hot encoded with missing values as a separate category. Imputation and scaling were fitted inside each training fold, so held-out variants did not inform them. Every analysis can be rerun from Table S1 with the deposited scripts. Model 1b uses FoldX ΔΔG, DynaMut2 ΔΔG, GREMLIN-style ΔΔE, Combined_score (the sum of z-scored ΔΔE and FoldX ΔΔG), position entropy, ortholog conservation statistics, AlphaFold3 pLDDT, IUPred3 disorder, and PSIPRED confidence. It also uses categorical domain, secondary-structure, mutant-seen-in-orthologs, and interface-membership features. Model 1 adds REVEL, CADD, SIFT, PolyPhen, phyloP, and gnomAD allele frequency. The label is 1 for ClinVar pathogenic/likely pathogenic or a published pathogenic report and 0 for benign/likely benign (Table S3); the 13 post hoc additions were excluded by design. Performance was estimated by 5-fold stratified cross-validation (out-of-fold probabilities from cross_val_predict), with 95% bootstrap confidence intervals (2000 resamples). Residue-grouped cross-validation (StratifiedGroupKFold with residue position as the group, five seeds) kept all substitutions at a position in the same fold. Imbalance-aware metrics were precision–recall curves with each class as the positive class, balanced accuracy, the Matthews correlation coefficient, and confusion matrices at a 0.5 threshold, with Wilson intervals for recall. A second threshold was chosen inside each training fold by maximizing Youden’s J on inner out-of-fold predictions. Per-variant attributions were computed with SHAP 0.54.1 TreeExplainer [36] on the held-out fold and summed by evidence group. Ablations refit Model 1b on subsets of feature groups (five seeds, stratified and residue-grouped).

### 4.5. High-Confidence Label Subset

The high-confidence subset of the 311 labels required either a published functional-assay result concordant with the label or a ClinVar review status of two stars or more. Qualifying review statuses were ‘reviewed by expert panel’ and ‘criteria provided, multiple submitters, no conflicts’. A functional assay was used only to confirm a label, and a normal result in one assay was not accepted as evidence of benignity. Variants that are pathogenic in ClinVar but normal in one assay (R200G, D176Y) are therefore not benign-like in this subset. Both models were refit on this subset with bootstrap 95% confidence intervals. Functional-assay results were compiled from four published studies [37,38,39,40] and are listed in Table S12; no new experiments were performed for this study.

### 4.6. Multi-Seed Cross-Validation

Headline values use random_state = 42. Both models were also refit with 20 seeds (0–19; each seed controls the fold split and the forest), and the mean ± SD is reported.

### 4.7. Temporal Validation, DeLong and Equivalence Tests

Two temporal partitions were used. In the recent-reassessment holdout, each variant was dated by the year its ClinVar classification was last evaluated or, without a ClinVar record, by the year of its first literature report. In the first-availability holdout, each variant was dated by the earlier of its first ClinVar evaluation year and its first literature report. Archived ClinVar releases were not used, so a variant submitted to ClinVar before 2025 but first evaluated and first published later could be placed in the test partition too late. Variants dated before 2025 formed the training partition and those dated 2025–2026 the test partition; undated variants were excluded. Paired DeLong tests compared each model with AlphaMissense on the same test variants. Equivalence was tested with two one-sided tests (TOST), using the DeLong standard error of the paired AUC difference and a pre-specified ±0.05 margin. Equivalence was declared if the 90% confidence interval of the difference lay within the margin.

### 4.8. Population-Frequency Sensitivity Analysis

VUS-like variants (excluding the 13 post hoc additions) with a gnomAD v4.1 allele frequency above a threshold were added to the benign class. Three thresholds were tested. The first, 1 × 10^−5^, is a lenient cut-off. The second, 1.4 × 10^−5^, is the maximum credible allele frequency from the standard formula [17], with prevalence 1/8000, maximum allelic contribution 0.1, and penetrance 0.9. A penetrance of 0.9 reflects the near-complete penetrance of HHT2 in adults while allowing for young gnomAD participants who may not yet have symptoms. Complete penetrance (1.0) gives a threshold of 1.25 × 10^−5^, adds one more variant (44 in total), and gives nearly the same result (Model 1b 0.941 ± 0.008; Table S7). The third, 8 × 10^−5^, is one tenth of the HHT VCEP BS1_Supporting threshold. The HHT VCEP thresholds (BS1_Supporting above 0.08% and BS1 above 0.2%, applied to the Popmax/Grpmax filtering allele frequency) [18] were also checked; no VUS-like variant reaches them. To look for benign variants outside the training set, all 605 ACVRL1 missense variants in gnomAD v4.1 (joint exome and genome data; grpmax FAF95) were screened against these thresholds. The All of Us Data Browser (286 missense variants) and the UK Biobank exome variant table in Genebass (200 variants) were screened as well. All three were accessed on 19 October 2026; the All of Us and Genebass views were limited to 200 rows each and are therefore incomplete. In gnomAD, seven variants met a VCEP frequency code (BS1: A482V and L162F; BS1_Supporting: T450A, R218W, L220F, S110L, and P26L), and none reached BA1. In UK Biobank, only A482V exceeded a threshold (overall allele frequency 0.48%). All seven were already benign-like training labels, so no labels were added. Documented ACVRL1 founder variants [41,42,43,44] do not approach a 10% share of pathogenic alleles, so the 0.1 contribution was retained. Raw overall allele frequencies were used because filtering allele frequencies and allele counts were not available for all variants. The highest raw frequency among the VUS-like variants is 2.2 × 10^−4^ (I417F), almost four-fold below the BS1_Supporting threshold. gnomAD allele frequency was removed from Model 1 for every expanded set to avoid circularity.

### 4.9. Structural Illustrations

The four worked-example residues were mapped onto the AlphaFold3 monomer. Cys34’s disulfide partner was identified by a pairwise Sγ–Sγ distance scan (Biopython 1.87) across all 18 cysteines (cut-off 3.0 Å); Cys34–Cys51 was the only pair involving Cys34. Solvent accessibility of Glu281 was computed with the Shrake–Rupley algorithm (Biopython). Figure 6 shows PyMOL 3.1.3 renderings of the four variants, generated by the accompanying script (worked_examples_pymol.pml): a whole-chain overview (A) and close-ups (B–E). Mutant side chains in Figure 6 are in-place PDBFixer 1.12.0 models shown for illustration only; the locally relaxed models (Section 4.2) are provided as File S1.

### 4.10. Benchmark Comparators

AlphaMissense scores [13] were obtained from the published proteome-wide prediction set. Rhapsody-2 [15] (github.com/anupam-banerjee/rhapsody-2) was cloned and run locally for all 886 variants in the original screen, including its evolutionary-conservation branch. For that branch, a BLASTP 2.17.0+ search (database clustered_nr, E-value threshold 0.001, query P37023) was submitted directly to NCBI. Its results were supplied to Rhapsody-2 in place of its own timeout-limited automated BLAST call, so the full dynamics-plus-conservation model could be run. Approximately one-fifth of the 886 screened variants (165) are plausibly present in Rhapsody-2’s own training set (Section 2.9); AUC figures restricted to the independent subset are reported alongside the all-variant figures for this reason. ESM1b and popEVE scores [45,46] were obtained via a bulk EBI ProtVar export. Rhapsody-2 full-model scores for all 886 variants are given in Table S11. For the matched comparison (Figure 7), all predictors were evaluated on the same labeled variants, using out-of-fold predictions for Models 1b and 1.

### 4.11. Evo2 Zero-Shot Genomic Foundation Model Comparison

Of the 899 variants in this dataset, 565 have both a confirmed GRCh38 (chr12) genomic coordinate and an unambiguous single-nucleotide reference/alternate allele parseable from their cDNA HGVS notation. These are the original 479-variant batch plus 86 *post hoc* literature-mined variants for which a genomic coordinate was subsequently resolved. The remaining 334 were excluded from this comparison rather than assigned an inferred coordinate. Of these, 319 had only a genomic-position-derived identifier and no parseable cDNA HGVS substitution, and 13 were *post hoc* literature-mined additions with no Variant_ID recorded. The other two had a valid cDNA HGVS substitution, but their genomic coordinate could not be confirmed. ACVRL1 lies on the plus strand of chromosome 12 (Ensembl gene ENSG00000139567, confirmed via the Ensembl REST API), so cDNA-derived reference/alternate alleles map onto the GRCh38 sequence without reverse-complementation. This was cross-validated by confirming the expected reference base at all 565 genomic positions against the fetched GRCh38 sequence (zero mismatches). For each variant, an 8192 bp reference window (taken from GRCh38 chr12:51,908,379–51,924,976, fetched via the Ensembl REST API) was centered on the variant position, with 4096 bp of context on each side. This matches the window size and centering used in Evo2’s own published BRCA1 zero-shot variant-effect example. A corresponding variant window was constructed by substituting the single alternate allele. Both windows were submitted to Evo2-7B’s hosted forward-pass inference endpoint (NVIDIA NIM, health.api.nvidia.com; unembed output layer), which returns per-position next-nucleotide logits. A teacher-forced sequence log-likelihood was computed by summing the log-softmax probability of each actual next base across the full window. The delta score (variant minus reference log-likelihood) was used as the variant-effect estimate, with no ACVRL1- or HHT-specific fine-tuning of Evo2 at any stage [21]. Table S1 reports Evo2 scores for these 565 variants only.

## 5. Conclusions

For ACVRL1, a classifier built from evolutionary conservation and protein-stability estimates performs close to AlphaMissense in discriminating pathogenic from benign missense variants. Unlike AlphaMissense, it also shows the structural or evolutionary evidence behind each call, which turns a score into a testable hypothesis about mechanism. Its main weakness is the recognition of benign variants. Formal equivalence with AlphaMissense was supported only for the companion model that also uses sequence-based predictors. Every training label is traceable to a ClinVar record or a publication, and the code, labels, and out-of-fold predictions are deposited for reuse. The classifier’s most realistic near-term use is to prioritize VUS for functional testing, not to determine clinical pathogenicity on its own. Functional testing of discordant calls such as E281Q is the most direct way to test whether the per-variant attribution is useful.

## Figures and Tables

**Table 1:** Imbalance-aware performance on the 311 labeled variants (out-of-fold predictions for Models 1b and 1; 95% bootstrap CI for ROC-AUC). Benign PR-AUC treats the benign class as positive (baseline 0.05). Results at a threshold chosen inside each training fold are given in the text and Table S4.

MCC	Balanced Accuracy	Pathogenic Correct	Benign Correct	Benign PR-AUC	ROC-AUC (95% CI)	Threshold	Predictor
0.51	0.81	283/296	10/15	0.62	0.954 (0.916–0.984)	0.5	Model 1b
0.65	0.85	289/296	11/15	0.66	0.979 (0.957–0.994)	0.5	Model 1
0.55	0.92	271/296	14/15	0.82	0.983 (0.962–0.997)	0.564	AlphaMissense

**Table 2:** Paired comparisons with AlphaMissense on the two temporal holdouts (final 311-variant label set). DeLong *p* tests for a difference in ROC-AUC; TOST *p* tests equivalence within ±0.05 AUC (*p* < 0.05 supports equivalence). Results on the high-confidence label set are in Table S5.

TOST *p*	DeLong *p*	AlphaMissense AUC	Model AUC	N Test (P/B)	Model	Holdout
0.002	1.00	0.992	0.992	35 (31/4)	Model 1 (full evidence)	First availability
0.069	0.48	0.992	0.976	35 (31/4)	Model 1b (structural/conservation)	First availability
<0.001	0.74	0.997	0.994	72 (67/5)	Model 1 (full evidence)	Recent reassessment
0.099	0.35	0.997	0.976	72 (67/5)	Model 1b (structural/conservation)	Recent reassessment

**Table 3:** Sensitivity of the classifiers to adding population-frequency-defined benign variants to the final 311-variant label set (5-fold CV ROC-AUC, mean ± SD over five seeds; 20 seeds for the unexpanded set). Benign variants added: VUS-like variants without a training label (excluding the 13 post hoc additions) whose gnomAD v4.1 allele frequency exceeds the threshold. Each lower threshold includes the variants of the higher ones. gnomAD allele frequency was removed from Model 1 for all expanded sets. Residue-grouped values are in Table S7.

8 × 10^−5^	1.4 × 10^−5^	1 × 10^−5^	None (Final Labels)	Benign Threshold (gnomAD AF)
5	43	50	0	Benign variants added
0.917 ± 0.003	0.937 ± 0.006	0.937 ± 0.006	0.947 ± 0.008	Model 1b ROC-AUC
0.972 ± 0.004	0.981 ± 0.002	0.982 ± 0.001	0.976 ± 0.004	Model 1 ROC-AUC

**Table 4:** Summary of the two classifiers and the comparator predictors. ROC-AUC is given on the 311 labeled variants (out-of-fold for Models 1b and 1; REVEL and CADD cover only 41 of them) and, in parentheses, on the 39 variants scored by all predictors (Figure 7).

Suggested Use	Main Limitation	Evidence Shown Per Variant	ROC-AUC	Inputs	Predictor
Mechanistic hypotheses; choice of functional assay	Benign recall 10/15; ACVRL1 only; not calibrated	Yes, by evidence group	0.954 (0.903)	Conservation, ΔΔG and structural context; no population data	Model 1b
Research use	Possible circularity through REVEL and allele frequency; not calibrated	Partly	0.979 (0.925)	Model 1b features plus REVEL, CADD, SIFT, PolyPhen, phyloP and allele frequency	Model 1
Genome-wide computational evidence (PP3/BP4)	No per-variant rationale	No	0.983 (0.935)	Protein language model with structural context and population data	AlphaMissense
PP3/BP4 at ClinGen-calibrated thresholds [22]	Low coverage of these variants; REVEL trained on HGMD data	No	0.836, 0.868 (0.828, 0.847)	Ensemble and integrative scores	REVEL, CADD
Independent mechanistic comparator	165 ACVRL1 variants in its training data	Partly, by feature class	0.904 (0.802)	Dynamics, structure and coevolution	Rhapsody-2
Research use	Needs genomic coordinates; lowest accuracy here	No	0.797 (0.708)	Genomic language model, zero-shot	Evo2

**Table 5:** Structure used for each structure-based calculation.

Used in	Structure	Calculation
Models 1 and 1b	AlphaFold3 monomer (repaired)	FoldX ΔΔG
Models 1 and 1b	PDB 6SF3 chain A (extracellular), 3MY0 chain A (kinase)	DynaMut2 ΔΔG
Models 1 and 1b	AlphaFold3 monomer	pLDDT, DSSP secondary structure
Model 1b	AlphaFold3 complex models	BMPR2/FKBP1A interface membership (PISA-style)
Ablation only (Table S4); not in models	AlphaFold Database model (P37023)	OpenMM molecular dynamics (exploratory)
Section 2.7, File S1	AlphaFold3 monomer	Local relaxation of worked examples
Section 2.6 and Section 2.9	ESMFold model (pTM 0.643)	Structural cross-check, PAE (Figure 4), Rhapsody-2

## Data Availability

The full variant-level dataset (899 ACVRL1 missense variants with all computational, literature-derived, and model-score features) accompanies this submission as a Supplementary zip file. The zip file also contains the training labels with their sources, out-of-fold and holdout predictions, both GREMLIN-style alignments, and all analysis and figure-generation scripts. All files are available at Zenodo (doi: 10.5281/zenodo.23534587).

## Supplementary Materials

The following supporting information can be downloaded at: https://www.mdpi.com/article/10.3390/ijms27219742/s1.
